# IgG4-related hypophysitis: a retrospective cohort study

**DOI:** 10.1007/s00701-022-05231-9

**Published:** 2022-05-07

**Authors:** R. Bhargava, Z. Hussein, N. L. Dorward, J. P. Grieve, Z. Jaunmuktane, H. J. Marcus, I. Proctor, S. E. Baldeweg

**Affiliations:** 1 Department of Endocrinology, University College London Hospitals NHS Foundation Trust, London, UK; 2 Centre for Obesity & Metabolism, Department of Experimental and Translational Medicine, Division of Medicine, University College London, London, UK; 3 Department of Neurosurgery, National Hospital for Neurology and Neurosurgery, University College London Hospitals NHS Foundation Trust, London, UK; 4 Division of Neuropathology, National Hospital for Neurology and Neurosurgery, University College London Hospitals NHS Foundation Trust, London, UK

**Keywords:** Hypophysitis, Pituitary biopsy, IgG4, Hypopituitarism

## Abstract

**Purpose:**

IgG4-related hypophysitis (IgG4-RH) is a rare chronic inflammatory condition of the pituitary gland. This study reports the presentation, management and outcomes for patients with histologically proven IgG4-related hypophysitis.

**Methods:**

A prospectively maintained electronic database was searched over a 14-year period from 1 January 2007 to 31 December 2020 at a single academic centre to identify all patients with a histological diagnosis of IgG4-RH. A retrospective case note review from electronic health records was conducted for each case to extract data on their presentation, management and outcomes.

**Results:**

A total of 8 patients (5 male) with a median age of 51 years were identified. The most common presenting symptoms were headache (4/8; 50%), fatigue (3/8; 37.5%) and visual impairment (2/8; 25%). Three patients were initially treated with high-dose steroids aiming for reduction of the pituitary mass. However, ultimately all patients underwent transsphenoidal surgery. Post-operative changes included radiological reduction in pituitary mass in all patients that had imaging (7/7; 100%), improvement in vision (1/2; 50%), residual thick pituitary stalk (5/7; 71.4%), persistent anterior hypopituitarism (4/8; 50%) and panhypopopituitarism including diabetes insipidus (3/8; 37.5%).

**Conclusions:**

IgG4-RH is an increasingly recognised entity presenting with a variety of symptoms and signs. Clinical presentation is similar to other forms of hypophysitis. It is therefore important to consider IgG4-RH as a differential and to have a low threshold for pituitary biopsy, the diagnostic gold standard. The diagnosis of IgG4-RH will guide decisions for additional workup for IgG4-related disease, multi-disciplinary team involvement and follow-up.

## Introduction

IgG4-related disease (IgG4-RD) is an incompletely understood immunological condition that can affect various organs in the body [31]. A relatively new disease, it was first associated with autoimmune pancreatitis in 2001, and its extra-pancreatic manifestations were recognised in 2003 [23]. Histologically proven pituitary disease was first described in 2007 [36]. Although IgG4-RD is rare, it is even more unusual to find isolated pituitary gland manifestations, termed IgG4-related hypophysitis (IgG4-RH).

Inflammation of the pituitary gland (hypophysitis) is rare with a reported incidence of around 1 per 9 million population [6] and a prevalence of 0.2–0.88% of all pituitary surgery cases [13, 21].

Hypophysitis can be classified in multiple ways: by anatomical location of pituitary involvement, by aetiology and by pathological examination. The anatomical classification serves to inform the extent of involvement of the pituitary gland and its associated hormone deficiencies. It either involves the adenohypophysis, neurohypophysis including the stalk or both. Aetiological classification is either primary or secondary hypophysitis. Primary hypophysitis includes cases with a distinct autoimmune mechanism or idiopathic in nature. Secondary causes include those with a clear aetiopathogenesis often due to inflammation secondary to other sellar diseases or part of multi-organ systemic involvement. Pathological variants include lymphocytic or lymphoplasmacytic with or without sclerosis, non-necrotizing granulomatous and necrotizing granulomatous.

IgG4-RH is reported in fewer than 5% of all cases of hypophysitis [10]. However, its prevalence could be higher as a recent study reported that previously diagnosed lymphocytic hypophysitis cases or unspecified hypophysitis cases were in fact IgG4-RH on repeat pathological examination [4]. It is the only form of hypophysitis which has its own clinical diagnostic criteria as proposed by Leporati et al. in 2011[26], though there are distinct pathological diagnostic criteria for all known subtypes. Two systematic reviews have described the clinical characteristics of all the known cases of IgG4-RH. We aim to report a single-arm retrospective cohort review of all patients with a definitive histopathological diagnosis of IgG4-RH at our centre [1, 27].

## Methods

This is a retrospective cohort study including all patients with IgG4-RH confirmed on histopathology. The Strengthening the Reporting of Observational Studies in Epidemiology (STROBE) statement was used in the preparation of this section of the manuscript [32]. This study was registered as a service evaluation study with the University College London Hospitals NHS Foundation Trust Clinical Audit Committee.

### Study design and population

The study was conducted at the National Hospital for Neurology and Neurosurgery (NHNN), which is a tertiary referral centre and the largest pituitary centre in the UK [18]. Management for each case was discussed in our multi-disciplinary team meeting including endocrinologists, neurosurgeons, neuropathologists, neurooncologists and neuroradiologists. Surgery was recommended in patients for several reasons, including clinical visual impairment, radiological compression of the optic apparatus, diagnostic uncertainty and, in our cohort, failure of glucocorticoids to resolve presenting symptoms. Surgery was performed using a transsphenoidal approach, by three experienced neurosurgeons using either an operating microscope (JPG) or endoscope (HJM and NLD). Care was taken to debulk the mass to reduce pressure on the optic apparatus, and abnormal tissue was taken for diagnosis. Following surgery, each case was rediscussed in the same multi-disciplinary team meeting (MDT) to confirm pathological findings, clinical progress and radiological changes. All cases are kept in a prospectively maintained database. We searched this database over a 14-year period from January 1, 2007 to December 31, 2020, to identify all cases of IgG4-RH. These dates align with the earliest retrospective data available and when the study was registered respectively.

### Variables and data sources

A retrospective case note review from electronic health records was conducted for each case to extract data on patients’ demographics, clinical presentation, endocrine assessment, radiological morphology, histology and treatment outcome.

We included only patients with histological evidence of IgG4-RH. All patients therefore had undergone transsphenoidal surgery. Routine screening for IgG4-RH at NHNN started in 2011, when assays to measure serum IgG4 were available to the pathology service and all hypophysitis cases going back to 2007 have been subsequently reviewed, screened and pathological specimens re-stained by immunophenotyping for IgG4-related disease.

Data collection on presentation included age, sex, ethnicity, visual and endocrinological outcomes, imaging features and serum IgG4 levels. Radiological data were recorded from pre-operative pituitary magnetic resonance imaging (MRI) scans including size of the pituitary mass, enhancement of mass, thickness of stalk and optic chiasmal compression. Data included pituitary hormone profile, levels of follicle-stimulating hormone (FSH), luteinizing hormone (LH), thyroid-stimulating hormone (TSH), free thyroxine (fT4), prolactin, insulin-like growth factor-1 (IGF-1) and 9 am cortisol. Patients were considered to have adult growth hormone deficiency if there was failure of GH response (< 3 μg/L) on dynamic testing by an insulin tolerance test or glucagon stimulation test post-operatively. Adrenocorticotropic hormone (ACTH) deficiency or secondary adrenal insufficiency was defined as low morning cortisol (< 100 nmol/L), an insufficient serum cortisol response during standard dynamic testing as above or if the patient was receiving glucocorticoid replacement therapy pre-operatively. Gonadotrophin deficiency was considered in men with low morning serum testosterone levels, pre-menopausal women with low serum estradiol with inappropriately normal or low LH and FSH levels and post-menopausal women with inappropriately normal or low LH and FSH levels. TSH deficiency was considered with low or normal TSH levels and low fT4 levels. Central diabetes insipidus was diagnosed biochemically in the context of polyuria and polydipsia or in those in those cases where desmopressin was started for their osmotic symptoms pre-operatively. Data on treatment included high-dose steroids, surgical resection and the use of immunosuppressive drugs. Data of pathology included histological examination to look for IgG4 plasma cells per high power frame, their ratio with total IgG cells and correlation with biochemical serum IgG4 levels.

Outcome data reported endocrine and radiological outcome. Endocrine outcome was based on baseline pituitary profile testing and where applicable on dynamic functional testing. Radiological follow-up was based on MDT recommendation and included serial interval scans. The focus of the scans was to look for any residual mass, reduction in size, change in thickness of stalk and persistence of optic chiasmal compression.

### Study size and statistical methods

No formal power calculation was performed. Due to limited incidence of the disease, we included all cases that were histologically diagnosed at our centre between 2007 and 2020. Basic data was evaluated using descriptive statistics. Mean and standard deviation (SD) were used to describe continuous variables. Median and interquartile range (IQR) were used to describe data not normally distributed.

## Results

### Presentation

Over the 14-year span, we identified 40 cases that had a surgical biopsy to determine the cause of hypophysitis. IgG4-RH was detected in a total of eight patients (5 male with a median age of 51 years, IQR (38–61.5) at diagnosis. The male-to-female ratio was 1.66. Majority of patients were Caucasian (5/8; 62.5%), followed by Asian (2/8;25%) and Black (1/8;25%) ethnicity. The most common symptom at presentation was headache (4/8; 50%), followed by fatigue (3/8; 37.5%) and clinical visual impairment (2/8; 25%). Symptoms related to endocrine dysfunction included sexual dysfunction (3/8; 37.5%) and osmotic symptoms of polyuria and/or polydipsia (3/8; 37.5%) (Table 1).

**Table 1:** Patient demographics, clinical presentation, treatment and endocrine status before and after surgery. F: female. M: male

Case	Age (years)	Sex	Ethnicity	Presenting symptoms	Pre-surgery endocrine status	Received steroids	Indication(s) for surgical intervention	Post-surgery endocrine status
1	44	F	Black	Headaches	Hyperprolactinemia	No	Diagnostic uncertainty	Lost to follow-up
2	26	M	Asian	Headaches, erectile dysfunction, fatigue and polyuria	Panhypopituitarism	No	Diagnostic uncertainty	Panhypopituitarism
3	60	M	White	Headaches, polyuria and polydipsia	Panhypopituitarism	No	Diagnostic uncertainty	Panhypopituitarism
4	74	M	White	Bitemporal hemianopia and collapse	Anterior hypopituitarism	4 weeks of prednisolone 20 mg and then post-operative intravenous methylprednisolone 1 g for 5 days and 4 weeks prednisolone 20 mg	Failure of glucocorticoids Clinical visual impairment	Anterior hypopituitarism
5	36	M	White	Headaches, infertility and lethargy	Anterior hypopituitarism	No	Diagnostic uncertainty Radiological compression of optic apparatus	Anterior hypopituitarism
6	58	M	White	Tired and reduced libido	Anterior hypopituitarism	No	Diagnostic uncertainty	Anterior hypopituitarism
7	62	F	White	Polyuria, polydipsia and headaches	Panhypopituitarism	Prednisolone 40 mg in reducing doses over 8 weeks	Failure of glucocorticoids Diagnostic uncertainty	Panhypopituitarism
8	44	F	Asian	Visual deterioration	Central hypothyroidism	Prednisolone 40 mg reducing over 6 months and azathioprine	Failure of glucocorticoids Clinical visual impairment	Anterior hypopituitarism

### Investigation

Endocrine assessment prior to surgical intervention determined that all patients had some degree of endocrine impairment. Anterior hypopituitarism was present in 7/8, 87.5% of cases. The most common deficiencies included TSH (7/8; 87.5%), ACTH (6/8; 75%), FSH and LH (6/8; 75%). Only one case (case 3) had an inadequate response of GH on insulin tolerance testing. Case 1 was not assessed for GH deficiency as was lost to follow-up. The other six cases did not have dynamic testing for GH deficiency but had low–normal IGF-1 levels with the presence of three or more pituitary hormone deficiencies to suggest they would have inadequate response to dynamic testing [19]. One case (case 1) only had hyperprolactinemia, three patients (cases 2, 3 and 7) had diabetes insipidus (diagnosed clinically and biochemically) and thus had panhypopituitarism (3/8; 37.5%).

**Fig. 1:**
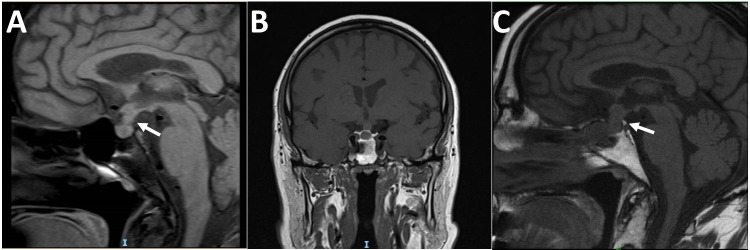
Post-contrast T1-weighted pituitary magnetic resonance (MR) images in one patient with IgG4-RH. **A** Pre-treatment sagittal section scan showing a pituitary adenoma 15 × 7 × 8 mm (height × width × length) with sellar extension (white arrow). Pituitary stalk is thick and bulges into infundibular recess, posterior bright spot of neurohypophysis is lost. **B** Post-treatment coronal section scan at 5 months after treatment with oral glucocorticoids over 4 weeks did not show any reduction in mass and visual loss persisted. **C** Post-operative sagittal section scan at 1 year following pituitary biopsy shows reduction in pituitary mass and residual thick pituitary stalk (white arrow)

Radiological imaging demonstrated features suggestive of hypophysitis including an enlarged pituitary mass in all patients with extension into the suprasellar space, a thickened pituitary stalk in 5 patients (5/8;62.5%) and one patient (case 5) had optic chiasmal compression (Table 2). The median duration of interval scans is 2.5 years IQR (1.5–4) after the surgical procedure. An enlarged pituitary gland with a thick stalk and loss of posterior bright spot is seen at presentation in Fig. 1A on pituitary MR (magnetic resonance) imaging.

**Table 2:** Features of magnetic resonance imaging of the pituitary gland for all patients. F: female. M: male. MRI: magnetic resonance imaging. FDG-PET: fluorodeoxyglucose-positron emission tomography; N/A: not available

Case	Pre-operative MRI				Post-operative surveillance imaging		
	Pituitary mass	Optic chiasmal compression	Thick stalk	Loss of posterior bright spot on T1 weighting	Reduction in pituitary mass on MRI	Thick stalk on MRI	FDG-PET
1	Present	Absent	No	No	N/A	N/A	N/A
2	Present	Absent	No	No	Yes	No	N/A
3	Present	Absent	Yes	No	Yes	Yes	Normal
4	Present	Absent	Yes	Yes	Yes	Yes	N/A
5	Present	Present	No	No	Yes	No	N/A
6	Present	Absent	Yes	No	Yes	Yes	Right submandibular gland avid; not IgG4
7	Present	Absent	Yes	No	Yes	Yes	N/A
8	Present	Absent	Yes	No	Yes	Yes	Non-specific enhancement of sella, low-grade uptake in large vessels

Only half (4/8; 50%) of the patients had initial biochemical evaluation for IgG4 levels as the test was not routinely done prior to 2011. The median level was 152.5 mg/dL (normal < 140 mg/dL), (Table 3).

**Table 3:** Biochemical and pathological findings. hpf: high power field; N/A: not analysed; F: female. M: male

Case	Histological IgG4/IgG ratio (normal < 40)	Histological IgG4 cells/hpf (normal < 10 cells/hpf)	Serum IgG4 levels (normal 0–140 mg/dL)	Histological features	Numeric weightage (ACR/EULAR criteria for IgG4-RD) ≥ 20 is diagnostic
1	40	65	N/A	Plasma cell-rich areas with fibrosis and lymphoid aggregates	20
2	25	35	51	Fibrosis and focal plasma cell infiltrate	24
3	30	20	N/A	Fibroconnective tissue containing areas of sclerosis and a dense inflammatory cell infiltrate composed of small lymphocytes, abundant histiocytes and plasma cells	20
4	80	150	500	Fragments of fibrous tissue showing a mixed chronic inflammatory cell infiltrate composed of histiocytes, small lymphocytes and plasma cells	35
5	50	80	N/A	Dense mixed inflammatory infiltrate comprising numerous lymphocytes and plasma cells	18 (No additional points gained for serum IgG4 levels as N/A)
6	50	80	108	Dense polymorphic infiltration composed of plasma cells, plasmacytic cells and small lymphocytes	22
7	20	10	N/A	Fibrous tissue with a lymphoplasmacytic inflammatory cell infiltrate	20
8	40	200	197	Patchy but dense plasma cell infiltrate	22

### Treatment and outcomes

Postoperative endocrine status after 1 year was as follows: anterior hypopituitarism (4/8; 50%), followed by panhypopopituitarism (3/8; 37.5%). One patient was lost to follow-up. Three patients were initially treated with high-dose glucocorticoids aiming for a reduction in the size of the pituitary mass. In these three cases (cases 4, 7 and 8) initially, oral glucocorticoid dose ranged between 20 and 40 mg of prednisolone for a minimum of 4 weeks followed by gradual dose reduction over a period of 1 to 6 months. In one patient (case 4), a short course of intravenous methylprednisolone was also trialed. However, despite relatively high-dose glucocorticoids, all patients proceeded to transsphenoidal surgery to make a definitive diagnosis. A persistently enlarged pituitary gland following treatment with oral glucocorticoids is seen in Fig. 1B.

Following a histological diagnosis of IgG4-RH, three asymptomatic patients from our case series (3/8; 37.5%) were screened to determine if their pituitary impairment was secondary to IgG4-RD. Fluorodeoxyglucose-positron emission tomography (FDG-PET) scans were performed to look for any extra-pituitary involvement: one patient (case 3) had a normal scan; one patient had an avid right submandibular gland (case 6), which was resected but with negative IgG4 histology; and the last patient (case 8) had non-specific activity in the pituitary gland and low-grade uptake in their large vessels. The remaining five asymptomatic cases did not have any imaging modality to screen for secondary involvement from IgG4-RD.

Postoperative scanning demonstrated radiological reduction in size of the pituitary mass in all patients, except (case 1) the one lost to follow-up. However, a residual thick pituitary stalk was seen in more than two-thirds of the patients (5/7; 71.4%). Of the two patients (2/8; 25%) that presented with clinical visual impairment (cases 4 and 8), there was improvement of visual outcomes in one of them. A residual thick stalk is seen in Fig. 1C, 1 year after surgical intervention.

All the cases were histologically confirmed to have IgG4-RH by immunophenotyping. The criteria for histopathologic diagnosis are in accordance with the Leporati criteria for diagnosing IgG4-RH, formulated in 2011 (described in “Discussion” section). In all patients, the number of IgG4 cells was ≥ 10/high power field (hpf). All cases met the numeric weightage criteria to be diagnosed as IgG4-related disease using the latest classification system as devised by the ACR/EULAR (American College of Rheumatology and the European League Against Rheumatism) in 2019 [33] (apart from case 5, who narrowly missed the cutoff due to not having serum IgG4 levels measured at diagnosis). Although this classification system does not include the pituitary gland as it focuses on 11 organs that are more frequently involved, it shows that patients with isolated pituitary disease would score highly on a composite of histopathological, immunostaining and serum IgG4 levels to be diagnosed as IgG4-RD only involving the pituitary.

## Discussion

### Principal findings

To our knowledge, this is the largest series of patients with a histological diagnosis of isolated IgG4-RH from a single centre and adds to the recent reviews of all cases described by Li et al. and Amirbaigloo et al. [1, 27]. The clinical findings of IgG4-RH are indistinguishable from other causes of hypophysitis. This incompletely understood disease needs to be considered as a differential diagnosis in all cases of hypophysitis. Our analysis showed that the median age for diagnosis is in the sixth decade of life. A trial of glucocorticoids should be attempted, if possible, as it typically leads to a reduction in pituitary mass. Use of high-dose glucocorticoids in three of our cases (cases 4, 7 and 8) without sufficient response ultimately led to surgical resection to relieve compressive symptoms and to diagnose IgG4-RH. Neurosurgical intervention indications are the same as for any pituitary mass: visual impairment, radiological compression of the optic apparatus and diagnostic uncertainty. However, IgG4 disease does not always respond to steroids [7, 25, 35]. Follow-up should include at least a yearly endocrine assessment as all the cases described had pituitary hormone deficiency after surgery (except one, who was lost to follow-up).

### Comparison with current literature

Our analysis shows that IgG4-RH presents in the sixth decade of life. It is associated with a 1.66:1 male preponderance. This is similar to the findings of the reviews by Li et al. and Amirbaigloo et al. This is different to other forms of hypophysitis which typically affect younger patients with a female preponderance.

The clinical presentation of IgG4-RH is principally divided into either endocrine dysfunction causing hypopituitarism, mass effects around the pituitary gland or a combination of both. The most commonly reported presentation is mass effects causing headaches followed by visual disturbance [12, 24] possibly due to stretching of the dura mater and possible inflammatory changes and abutting the optic chiasm respectively. These reports are in keeping with our cohort where headaches followed by fatigue and visual disturbance were the most common symptoms at presentation. There is no fixed pattern for hypopituitarism in IgG4-RH unlike that typically seen with pituitary adenomas [16]. The most common pituitary deficiency is ACTH deficiency, followed by gonadotrophin and then secondary hypothyroidism [12]. Hence, clinical features are dependent on the specific hormonal deficiency. Fatigue, muscle weakness, polyuria, polydipsia, erectile dysfunction, irregular menses and infertility have been observed [1, 27]. In our cohort, the most common hormone deficiency was TSH followed by ACTH and then gonadotrophin deficiency. In our cases, post-operative endocrine function did not improve and all patients (except case 1; lost to follow-up) required pituitary hormone replacement therapy.

The radiological appearance of hypophysitis varies according to imaging modality used. Computed tomography (CT) scan of the head may show a hypodense pituitary mass with adjacent bony changes, but it may also be completely normal [29]. Hence, a dedicated MRI of the pituitary gland is the investigation of choice to characterise the gland and detect hypophysitis [8]. Features of hypophysitis on MRI might vary from asymmetrically homogenous dumbbell or triangular-shaped enlarged pituitary gland, thickening of the stalk which is not usually displaced and loss of the posterior pituitary bright spot typically seen in patients presenting with central diabetes insipidus [8]. However, the strongest predictor of an inflammatory process along with the clinical information is stalk thickening [15]. Sometimes, an empty sella may also be found as a post-inflammatory response especially in ‘missed cases’ [17]. These missed cases which are part of the autoimmune hypophysitis spectrum may present as isolated hormone deficiencies with normal imaging. This is because of the tendency of the mass to shrink over time, as the inflammatory process resolves [20]. Our findings showed that the majority cases (5/8; 62.5%) had an enlarged pituitary gland with a thickened stalk.

We have used the criteria devised by Leporati et al. which are now widely accepted to diagnose IgG4-RH. It is currently the only pituitary specific clinical diagnostic criteria [26]. It includes 5 diagnostic criteria, and the diagnosis can also be made without a definitive histopathological examination.

Criterion 1: pituitary histopathology; mononuclear infiltration of the pituitary gland, rich in lymphocytes and plasma cells, with more than 10 IgG4-positive cells/hpf.

Criterion 2: pituitary MRI; sellar mass and/or thickened pituitary stalk.

Criterion 3: biopsy-proven involvement in other organs; association with IgG4-positive lesions in other organs.

Criterion 4: serology; increased serum IgG4 (> 140 mg/dL).

Criterion 5: response to glucocorticoids; shrinkage of the pituitary mass and symptom improvement with steroids.

Diagnosis of IgG4-RH is established when any of the following is fulfilled: Criterion 1 OR Criteria 2 and 3 OR Criteria 2, 4 and 5.

The ACR and EULAR have jointly set forward a point-based classification criterion for IgG4-related disease [33]. It has been developed using a large cohort and remains the latest diagnostic classification. Interestingly, this classification system although robust is not exhaustive, and it does not include the pituitary gland as IgG4-RH is atypical and infrequent. We have demonstrated (Table 3) that all our cases (except for case 5 with absent serum IgG4 levels) would meet the numeric criteria on histopathological, immunostaining and serum IgG4 levels alone.

Pathogenesis of the disease remains unclear; an autoimmune mechanism or chronic infection has been suggested as possible causes [37]. Autoantigen to proopiomelanocortin and growth hormone [22] have been suggested, and pituitary antibodies have been reported in some cases [14]. The normal pituitary gland does not contain any IgG4 plasma cells. It is important to be cautious to diagnose IgG4-RH solely on the biopsy [2] as IgG4 + cells may be seen in other conditions [14]. Serum IgG4 levels are often but not always elevated in IgG4-RH. They can also be elevated in other conditions [28, 30]. In our case series, we were only able to obtain data on serum IgG4 levels on half of the patients as routine measurement was started in 2011. It is not particularly useful as it is not specific [2], and there is no known correlation between the severity of the disease and serological levels [14]. There is also no role for serial measurements of serum IgG4 levels to monitor for disease activity [5].

There is no standardised treatment of IgG4-RH due to the rarity of the disease, varied clinical presentation and lack of clinical trials comparing the efficacy of different medical treatment options. The main aims of treatment are to reduce the mass effects and to compensate for pituitary hormone deficiencies. Typically, glucocorticoids are the first line treatment for autoimmune hypophysitis, and the dose is tapered over weeks to months guided by clinical manifestation and imaging [11]. Glucocorticoids were trialled in three of our patients (cases 4, 7 8) as there was no immediate need to intervene surgically; however, as there was no clinical improvement viz. no resolution in hemianopia and headaches and increased visual deterioration, they subsequently required surgery. In most recent reviews, about half of the patients had a pituitary biopsy to diagnose IgG4-RH and a further 30% were diagnosed following a biopsy of a separate site of disease, despite glucocorticoids being used to treat the disease [1, 27]. This also goes to show that one must consider IgG4-RH as a differential in all cases of autoimmune hypophysitis. The diagnosis has an impact on long-term management, because if glucocorticoids do not achieve remission, other promising steroid sparing agent may be tried and the diagnosis would also allow to screen for systemic involvement in other organs [3, 9, 25]. Isolated IgG4-RH was seen in 36% cases by Amirbaigloo et al. and in 65% from the German Pituitary Tumour Registry [34]. As with other case series, our cohort showed persistent pituitary hormone deficiencies on post-operative endocrinological testing [37].

Whilst there are established pathological diagnostic criteria for IgG4-RD, small biopsies lacking the characteristic morphological alterations or showing atypical histological features are not uncommon. It may still be difficult to diagnose confidently purely on pathological grounds, and correlation with clinical and serological findings allows a more confident diagnosis [2]. We strongly recommend a multi-disciplinary team approach to the management of IgG4-RH patients with radiologists, pathologists, neurosurgeons and endocrinologists. We suggest a national online registry to record data collection that will aid clinical studies with larger sample sizes.

## Limitations

This study has several limitations. As it represents a retrospective case series for a rare disease, the sample size is small. It is subject to selection bias, and being retrospective, it depends on the accuracy and availability of the data records. The diagnosis remains complex, and none of our patients had extra-pituitary involvement despite asymptomatic screening in several cases. Although this is a monocentric study given the multi-cultural patient base of the centre and uncertain guidance over a 14-year period, the management has not been homogenous. This cohort only includes patients with a confirmed histological diagnosis, and there are certainly more patients with IgG4-RH who did not require a surgical intervention. This limits its generalizability to larger populations of patients.

## Conclusions

**Fig. 2:**
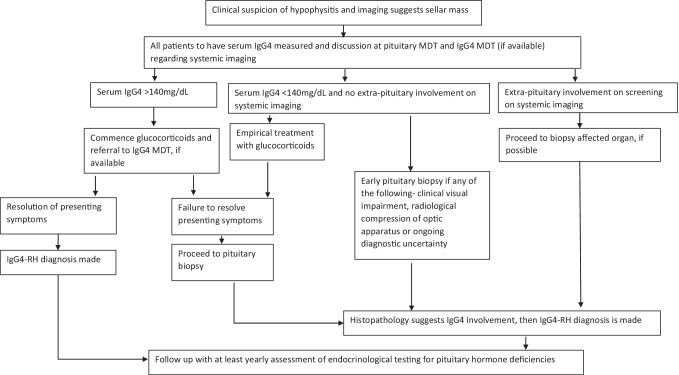
Algorithm depicting management of IgG4-related hypophysitis (IgG4-RH). *MDT* multi-disciplinary team meeting

IgG4-RH is an incompletely understood rare disease. As opposed to other causes of hypophysitis, which affect young female patients, IgG4-RH has a male preponderance in the sixth decade of life. It is being increasingly recognised due to greater awareness amongst physicians, greater access to the serum IgG4 assay and repeat staining of histological samples previously labelled with unspecified cause of hypophysitis. Given that there is a clinical overlap with other forms of hypophysitis and that the management is distinct, a low threshold should be kept for making a definitive diagnosis by a biopsy. We suggest that all patients should have a serum IgG4 measurement and discussion at the pituitary MDT and IgG4 MDT (if available) regarding extra-pituitary screening imaging if hypophysitis is suspected at diagnosis. If the serum IgG4 concentration is > 140 mg/dL and a trial of glucocorticoids resolve the presenting symptoms, then IgG4-RH is diagnosed according to the Leporati criteria. In case there is failure of resolution of presenting symptoms with glucocorticoids or if there is any surgical indication or diagnostic uncertainty and serum IgG4 concentrations are not elevated, then an early pituitary biopsy should be sought. If there is extra-pituitary organ involvement found on initial screening, then a biopsy of the affected organ should be sought to make a diagnosis of IgG4-RH. The approach to diagnosing and managing a case of suspected IgG4-RH is outlined in Fig. 2. Follow-up should include yearly endocrinological testing for pituitary hormone deficiencies.

## Data Availability

The data generated and analysed during the current study are available from the corresponding author on reasonable request.
